# Congenital thyroid hemiagenesis associated with follicular neoplasm: a case report

**DOI:** 10.1093/jscr/rjag441

**Published:** 2026-06-10

**Authors:** Loai Saleh Albinsaad, Fatimah A Aldarisi, Saud A Alwayil, Ali M Alsakkak, Nada I Almarzooq

**Affiliations:** Department of Surgery, College of Medicine, King Faisal University, Al-Ahsa City, Eastern Province 31982, Prince Faisal Bin Fahad Bin Abdulaziz Road, Al-Hofuf, Al-Ahsa, Saudi Arabia; College of Medicine, King Faisal University, Al-Ahsa City, Eastern Province 31982, Prince Faisal Bin Fahad Bin Abdulaziz Road, Al-Hofuf, Al-Ahsa, Saudi Arabia; College of Medicine, King Faisal University, Al-Ahsa City, Eastern Province 31982, Prince Faisal Bin Fahad Bin Abdulaziz Road, Al-Hofuf, Al-Ahsa, Saudi Arabia; College of Medicine, King Faisal University, Al-Ahsa City, Eastern Province 31982, Prince Faisal Bin Fahad Bin Abdulaziz Road, Al-Hofuf, Al-Ahsa, Saudi Arabia; College of Medicine, King Faisal University, Al-Ahsa City, Eastern Province 31982, Prince Faisal Bin Fahad Bin Abdulaziz Road, Al-Hofuf, Al-Ahsa, Saudi Arabia

**Keywords:** congenital anomalies, thyroid hemiagenesis, multi-nodular goiter, fine needle aspiration cytology, non-invasive follicular neoplasm

## Abstract

Thyroid hemiagenesis is a rare congenital anomaly characterized by the complete absence or underdevelopment of one thyroid lobe, resulting in the presence of only a single functional thyroid lobe. We present a unique case of a 34-year-old male patient who had congenital thyroid hemiagenesis and multi-nodular goiter. This case contributes to the existing medical literature by shedding light on the association between thyroid hemiagenesis and multi-nodular goiter. The patient experienced symptoms of right thyroid swelling and compression, and clinical examination revealed an enlarged right thyroid lobe with normal thyroid hormone and vitamin D3 levels. Ultrasound imaging showed an enlarged right lobe with multiple solid nodules, and fine needle aspiration cytology raised suspicion of a follicular neoplasm. Hemithyroidectomy confirmed the presence of a non-invasive follicular neoplasm. The case underscores the significance of comprehensive evaluation and surgical management in accurately diagnosing and treating thyroid hemiagenesis, which can be associated with many of thyroid disorders.

## Introduction

The absence of one thyroid lobe is the key characteristic of the rare congenital condition known as thyroid hemiagenesis (THA) [1]. Given the fact that the right lobe of the thyroid gland is often larger than the left in many people, THA might just be an exaggeration of this phenomenon [2].

Usually, the existence of this anomaly remains unnoticed and is found by incidence during the examinations carried out for other conditions [1]. Since the loss of one thyroid lobe typically does not result in clinical symptoms, the prevalence of THA is unknown; nonetheless, 329 instances documented between 1970 and 2010 demonstrate left lobe agenesis [2]. We present a unique case involving a euthyroid male patient who had left thyroid agenesis and a multi-nodular goiter on the right side who underwent right hemithyroidectomy as a part of the management.

## Case presentation

A 34-year-old Saudi male patient presented to the surgical clinic on 30/6/2020 with right thyroid swelling associated with compressive symptoms in form of dysphagia and dyspnea. Upon physical examination, the patient exhibited normal vital signs and was attentive. Upon thyroid examination, the right thyroid lobe was enlarged, with a firm, non-tender characteristic, the left side showed nodules whereas no discernible abnormalities.

Hormonal study and vitamin D3 level results were obtained as can be seen in Table 1. According to these results, the patient's thyroid function is within normal limits, indicating that the synthesis of thyroid hormones is not significantly aberrant.

**Table 1:** Lab results.

S.N.	Test name	Result	Normal range
1.	Free T4	16.99 pmol/L	10–25 pmol/L
2.	Free T3	4.90 pmol/L	2.8–7.1 pmol/L
3.	Thyroid stimulating hormone	1.96 uIU/mL	0.5–5.5 uIU/mL
4.	Vitamin D3	28.49 ng/mL	20–40 ng/mL

**Figure 1:**
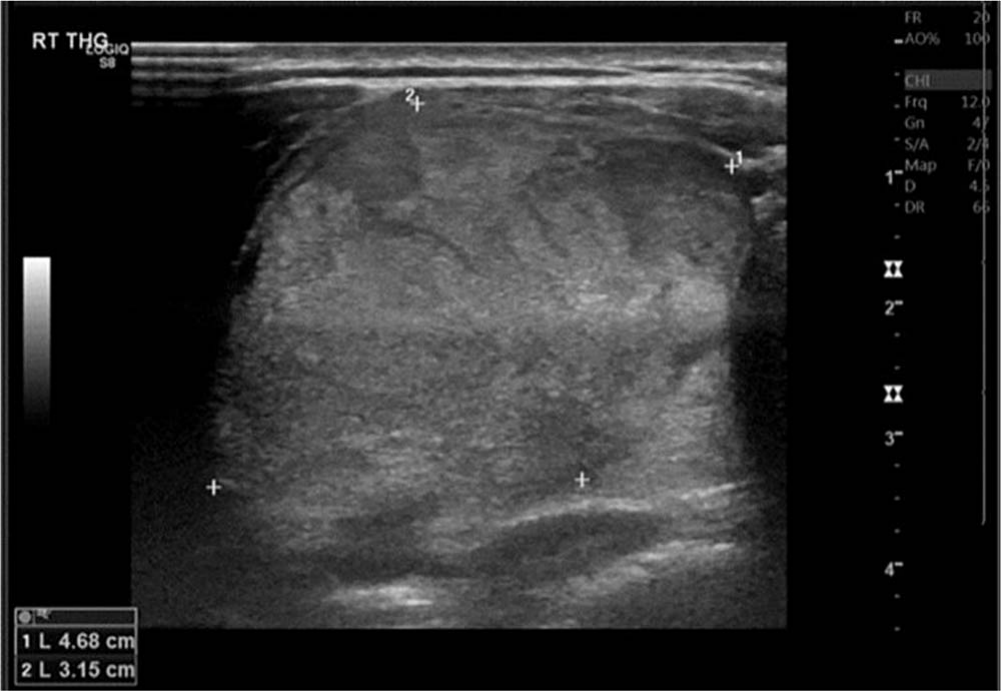
Thyroid ultrasonography reveals an enlarged right lobe of the thyroid.

**Figure 2:**
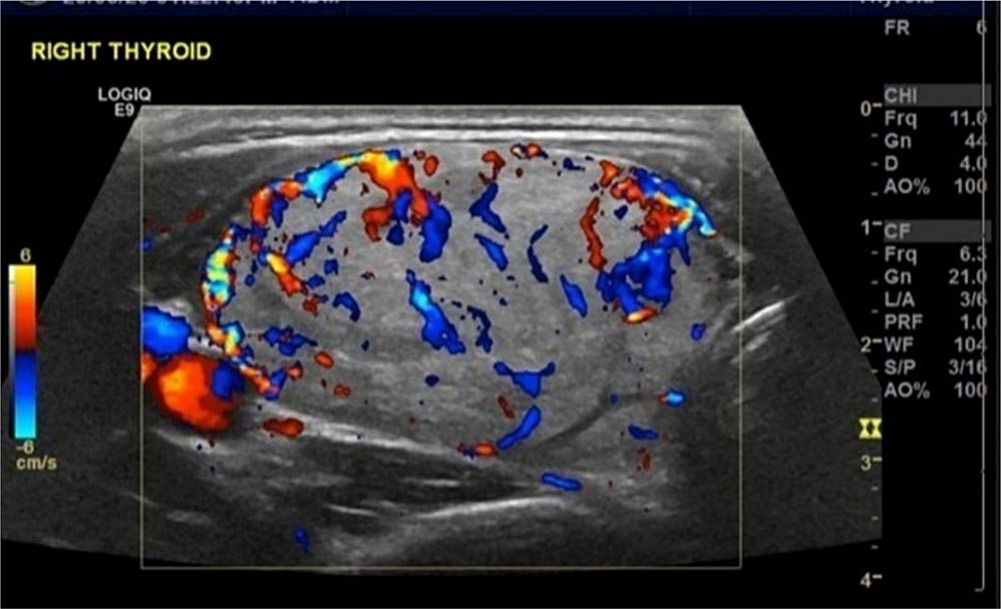
Thyroid color Doppler shows multiple oval isoechoic solid nodules are observed within the enlarged right thyroid lobe, with the largest measuring ~40 × 25 mm with perinodular vascularity.

**Figure 3:**
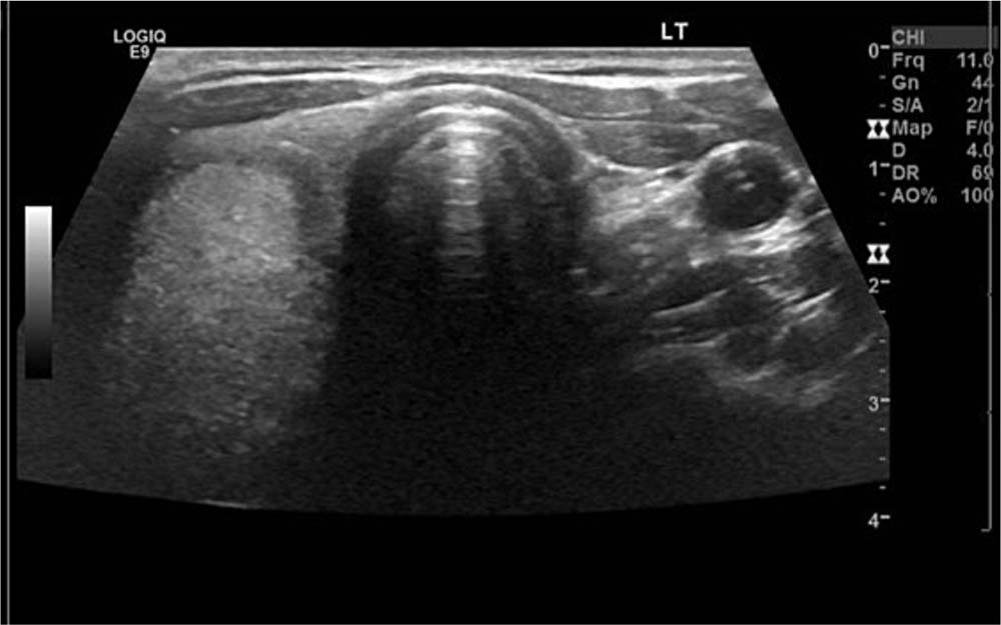
Ultrasound shows the left thyroid lobe is absent.

**Figure 4:**
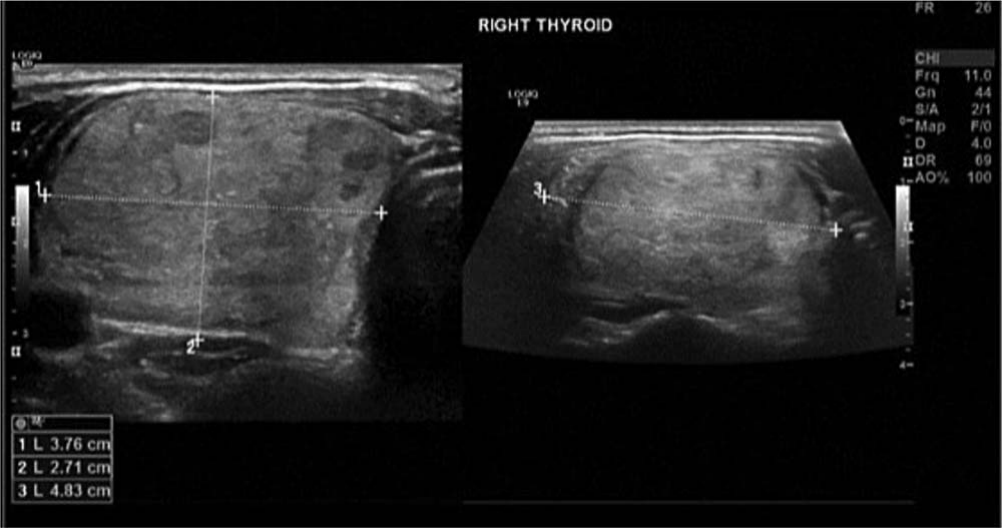
Ultrasound shows the dimensions of the right thyroid lobe measured at 38 × 27×48 mm.

Thyroid ultrasonography was conducted, and the findings revealed an enlarged right lobe of the thyroid (Fig. 1), showing multiple oval isoechoic solid nodules, with the largest measuring ~40 × 25 mm, accompanied by peri-nodular vascularity detected through color Doppler (Fig. 2). The left thyroid lobe was not visible (Fig. 3), while the dimensions of the right thyroid lobe were measured at 38 × 27 × 48 mm (Fig. 4). Additionally, non-specific cervical lymph nodes of small size were observed bilaterally.

Fine Needle Aspiration Cytology (FNAC) was performed by an interventional radiologist under ultrasound guidance, three passes of Fine needle aspiration (FNA) from Right thyroid nodule were done. After that, samples were labeled as 1-FNA without aspiration, and 2-FNA with aspiration. A total of 20 air-dried slides were received, along with a small amount of material for cell block preparation. Upon microscopic examination, the diagnosis is a suspicious follicular neoplasm in the right thyroid lobe nodule (BETHESDA IV).

Considering these findings, right hemithyroidectomy is advised for better assessment. Surgery was done and it was uneventful. A hemithyroidectomy specimen of the right lobe was received with the patient's name and medical record number. It consisted of a single piece of thyroid lobe that appeared nodular, firm, and encapsulated, measuring 6.5 × 4 × 4 cm in size. Upon cutting the surface, a nodule containing various sizes of cysts was observed. The nodule itself measured 4.5 × 4 × 4 cm, while the cysts exhibited diameters ranging from 0.2 to 0.4 cm.

The tumor was found to be unifocal, with the largest dimension measuring 4.5 cm in the right lobe. No other dimensions were identified. Histologically, the tumor was classified as a non-invasive follicular neoplasm with papillary-like nuclear features. No mitotic activity, tumor necrosis, angioinvasion (vascular invasion), lymphatic invasion, perineural invasion, capsular invasion, parathyroid gland, or psammoma bodies were identified in the specimen.

The diagnosis of left THA with right follicular neoplasm was made. The patient's recovery after the surgery was without any complications, and he was released from the hospital on the second day following the operation. He also did well during the 6-month follow-up.

## Discussion

THA is an unusual congenital abnormality that was first documented in the late 1800s. Previous data indicate that ˃50% of patients with THA also have concomitant thyroid illness [3]. These coexisting conditions may include a multinodular goiter [4–6], Graves’ disease [7], follicular neoplasm with Graves’ disease [8], hypothyroidism [9], or papillary thyroid carcinoma associated with Hashimoto's thyroiditis [10].

Underlying genetic aberrations have been found in some studies, the NKX2-5 gene has been found in two THA-afflicted siblings [11]. Another study involving two siblings found a correlation between the compound heterozygous mutations p.[Gly727Arg];[Gln1347Lys] of the GLI3 gene and the THA phenotype [12]. While the genetic predisposition has been found in a minority of cases, the pathogenesis of THA remains unclear. Additionally, important questions arise regarding the underlying mechanism of THA and its association with other thyroid disorders [1].

According to a narrative assessment, the majority of cases of left THA are predominantly female [3]. In contrast to this epidemiological role, we documented a male patient who had left THA. Due to the possibility of uncommon cases, it is imperative to evaluate patients of diverse genders with a high degree of suspicion.

It has been shown that THA frequently affects the left lobe of the thyroid gland [3]. Unlike the normal population, THA patients tend to have bigger size of the residual lobe. This can be attributed to a compensating mechanism attempting to prevent hypothyroidism [13, 14] consistent with the condition of our patient, who had an enlarged right thyroid lobe. Luckily, due to such compensation, the majority of THA patients exhibit euthyroid status throughout their lives [3]. Unfortunately, the continued compensatory mechanism and the state of elevated TSH (thyroid stimulating hormone) increases the risk for cancerous growth [15].

THA can be in many instances considered as only a benign congenital anomaly, as it can remain undiscovered and cause no symptoms in majority of the affected patients; and since the underlying mechanisms and clinical relevance of this anomaly are not fully established, well-defined management guidelines are absent, especially for patients who are asymptomatic [1]. In contrast, many patients undergo surgical removal of their contralateral lobe. This can be due to multiple reasons such as cosmetics, compression symptoms, or high risk of malignant transformation. In patients with thyroid hemiagenesis, surgical removal of the remaining lobe effectively constitutes a total thyroidectomy and necessitates lifelong thyroxine replacement. For those managed conservatively, regular monitoring of TSH and thyroxine levels is essential, as rising TSH may indicate compensatory changes or early neoplastic transformation [16].

The altered embryological development in THA creates uncertainty regarding parathyroid gland location, limiting the reliability of conventional anatomical expectations and complicating surgical planning [17]. Intraoperative identification of THA may reveal associated absence of ipsilateral parathyroid glands, further complicating dissection and increasing the risk of endocrine and nerve-related complications [18].

We emphasize the importance of multi-disciplinary teamwork to ensure a comprehensive assessment of the patient's condition. However, one of the limitations is the lack of genetic testing to detect putative underlying genetic abnormalities associated with multinodular goiter and THA. This case study highlights the importance of considering THA as an important factor that can be associated with many of thyroid disorders, especially in cases of asymmetric thyroid hypertrophy. For accurate diagnosis and treatment, a thorough diagnostic examination is essential. Thyroidectomy is a type of surgery that can work well in such cases. To fully understand its clinical consequences, further research is needed.

## Conclusions

This case highlights the clinical significance of thyroid hemiagenesis as a rare congenital anomaly that may coexist with other thyroid pathologies, including multinodular goiter and follicular neoplasms. While the condition itself does not require intervention, it is important to maintain a high index of suspicion in patients presenting with asymmetric thyroid enlargement, as coexisting pathology may necessitate surgical management—effectively resulting in total thyroidectomy and the need for lifelong hormone replacement therapy. Comprehensive diagnostic evaluation—including imaging, cytology, and histopathological assessment—is essential for accurate diagnosis and appropriate management. Additionally, altered anatomy and potential absence of parathyroid glands pose unique intraoperative challenges, emphasizing the importance of careful preoperative evaluation and surgical planning. Therefore, individualized, multidisciplinary decision-making is essential to balance the risks of surgery against the largely benign nature of this condition. This report contributes to the limited literature on the association between thyroid hemiagenesis and neoplastic conditions and reinforces the need for further research to better understand its pathogenesis and clinical implications.

## References

[1] Szczepanek-Parulska E, Zybek-Kocik A, Wartofsky L et al. Thyroid hemiagenesis: incidence, clinical significance, and genetic background. J Clin Endocrinol Metab 2017;102:3124–37. 10.1210/jc.2017-00784 28666345

[2] Sanctis VD, Soliman AT, Maio SD et al. Thyroid hemiagenesis from childhood to adulthood: review of literature and personal experience. *Pediatr Endocrinol Rev* 2016;13:499–506. 27116848

[3] Lesi OK, Thapar A, Appaiah NNB et al. Thyroid hemiagenesis: narrative review and clinical implications. *Cureus* 2022;14:e22401. 10.7759/cureus.22401 PMC8942040 35371763

[4] Bhartiya S, Verma A, Basu S et al. Congenital thyroid hemiagenesis with multinodular goiter. Acta Radiol Short Rep 2014;3:2047981614530286. 10.1177/2047981614530286 PMC4221940 25379177

[5] Aslaner A, Aydin M, Ozdere A. Multinodular goitre with thyroid hemiagenesis: a case report and review of the literature. Acta Chir Belg 2005;105:528–30. 10.1080/00015458.2005.11679774 16315840

[6] Karabay N, Comlekci A, Canda MS et al. Thyroid hemiagenesis with multinodular goiter: a case report and review of the literature. Endocr J 2003;50:409–13. 10.1507/endocrj.50.409 14599114

[7] Baldini M, Orsatti A, Cantalamessa L. A singular case of Graves’ disease in congenital thyroid hemiagenesis. Horm Res 2005;63:107–10. 10.1159/000084568 15775712

[8] Kim BK, Lee JW, Jung MJ et al. A case of thyroid hemiagenesis associated with Graves’ disease and follicular neoplasm. J Med Cases 2015;6:385–7. 10.14740/jmc2189w

[9] Shah RK, Bohara G, Juveria F et al. Hypothyroidism in thyroid hemiagenesis: a case report. Cureus 2022;14:e23087. 10.7759/cureus.23087 35464580 PMC9001868

[10] Alqahtani SM, Alanesi S, Alalawi Y. Thyroid hemiagenesis with primary hyperparathyroidism or papillary thyroid carcinoma: a report of two cases and literature review. Clin Case Reports 2021;9:1615–20. 10.1002/ccr3.3856 PMC7981713 33768901

[11] Szczepanek-Parulska E, Budny B, Borowczyk M et al. NKX2-5 variant in two siblings with thyroid hemiagenesis. Int J Mol Sci 2022;23:3414. 10.3390/ijms23063414 35328834 PMC8950672

[12] Szczepanek-Parulska E, Budny B, Borowczyk M et al. Compound heterozygous GLI3 variants in siblings with thyroid hemiagenesis. Endocrine 2021;71:514–9. 10.1007/s12020-020-02422-1 32696176 PMC7881956

[13] Suzuki S, Midorikawa S, Matsuzuka T et al. Prevalence and characterization of thyroid hemiagenesis in Japan: the Fukushima health management survey. Thyroid 2017;27:1011. 10.1089/thy.2016.0662 28657504 PMC5564018

[14] Mikosch P, Weixlbaumer V, Irrgang M et al. Hemiagenesis of the thyroid gland detected by coincidence-what is the clinical relevance? Case report and review of the literature. Wien Med Wochenschr 2020;170:403–9. 10.1007/s10354-020-00783-w 33026543 PMC7593389

[15] Haymart MR, Repplinger DJ, Leverson GE et al. Higher serum thyroid stimulating hormone level in thyroid nodule patients is associated with greater risks of differentiated thyroid cancer and advanced tumor stage. J Clin Endocrinol Metab 2008;93:809–14. 10.1210/JC.2007-2215 18160464 PMC2266959

[16] Sadhasivan L, Abhinaya R, Kumbhar US. Colloid nodular goitre associated with hemiagenesis of the thyroid gland. BMJ Case Rep 2024;17:e253843. 10.1136/BCR-2022-253843 PMC10806960 38199663

[17] Ferrari CC, Lorenz K, Dionigi G et al. Surgical strategy for primary hyperparathyreoidism with thyroid hemiagenesis. Langenbecks Arch Surg 2014;399:1077–81. 10.1007/S00423-014-1228-0 25078534

[18] Wang M, Hou L, Chen M et al. Thyroid hemiagenesis and Hashimoto’s thyroiditis—diagnostic and treatment pitfalls. *World J Surg Oncol* 2017;15:182. 10.1186/s12957-017-1250-0 PMC6389105 28985747

